# Supplementary material for: Risk factors for sacrococcygeal pilonidal sinus: a systematic review and meta-analysis supplemented by genetic causal assessment
Source: Front Surg. 2026 Jan 7;12:1718589. doi: 10.3389/fsurg.2025.1718589 (PMC12819706; doi:10.3389/fsurg.2025.1718589)
Supplement: Supplementary file 2 [file Datasheet2.zip › Supplementary Data 2/MR_pipeline_p5e-8/leaveone_plots/ukb-a-224.finngen_R12_L12_PILONIDALCYST.pdf]

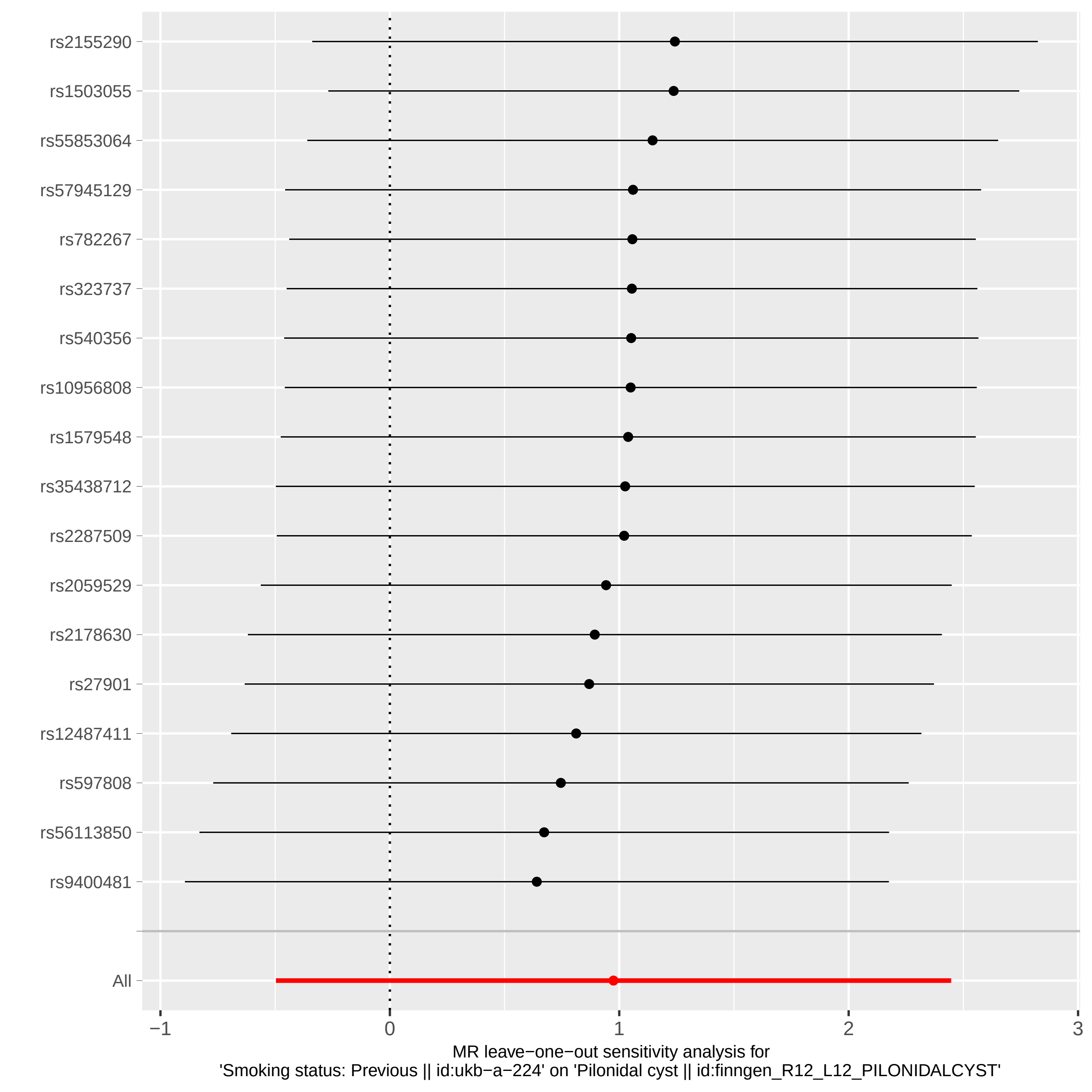
rs2155290
rs1503055
rs55853064
rs57945129
rs782267
rs323737
rs540356
rs10956808
rs1579548
rs35438712
rs2287509
rs2059529
rs2178630
rs27901
rs12487411
rs597808
rs56113850
rs9400481
All
−1
0
1
2
3
MR leave−one−out sensitivity analysis for
'Smoking status: Previous || id:ukb−a−224' on 'Pilonidal cyst || id:finngen_R12_L12_PILONIDALCYST'
